# Dissecting the dominant hot spring microbial populations based on community-wide sampling at single-cell genomic resolution

**DOI:** 10.1038/s41396-021-01178-4

**Published:** 2021-12-30

**Authors:** Robert M. Bowers, Stephen Nayfach, Frederik Schulz, Sean P. Jungbluth, Ilona A. Ruhl, Andriy Sheremet, Janey Lee, Danielle Goudeau, Emiley A. Eloe-Fadrosh, Ramunas Stepanauskas, Rex R. Malmstrom, Nikos C. Kyrpides, Peter F. Dunfield, Tanja Woyke

**Affiliations:** 1 U.S. Department of Energy, Joint Genome Institute, Berkeley, CA USA; 2 Environmental Genomics and Systems Biology Division, Lawrence Berkeley National Laboratory, Berkeley, CA USA; 3 Department of Biological Sciences, University of Calgary, 2500 University Dr. NW, Calgary, AB T2N 1N4 Canada; 4 National Bioenergy Center, National Renewable Energy Laboratory, Golden, CO USA; 5 Bigelow Laboratory for Ocean Sciences, 60 Bigelow Drive, East Boothbay, ME USA

**Keywords:** Environmental microbiology, Microbial ecology, Population dynamics

## Abstract

With advances in DNA sequencing and miniaturized molecular biology workflows, rapid and affordable sequencing of single-cell genomes has become a reality. Compared to 16S rRNA gene surveys and shotgun metagenomics, large-scale application of single-cell genomics to whole microbial communities provides an integrated snapshot of community composition and function, directly links mobile elements to their hosts, and enables analysis of population heterogeneity of the dominant community members. To that end, we sequenced nearly 500 single-cell genomes from a low diversity hot spring sediment sample from Dewar Creek, British Columbia, and compared this approach to 16S rRNA gene amplicon and shotgun metagenomics applied to the same sample. We found that the broad taxonomic profiles were similar across the three sequencing approaches, though several lineages were missing from the 16S rRNA gene amplicon dataset, likely the result of primer mismatches. At the functional level, we detected a large array of mobile genetic elements present in the single-cell genomes but absent from the corresponding same species metagenome-assembled genomes. Moreover, we performed a single-cell population genomic analysis of the three most abundant community members, revealing differences in population structure based on mutation and recombination profiles. While the average pairwise nucleotide identities were similar across the dominant species-level lineages, we observed differences in the extent of recombination between these dominant populations. Most intriguingly, the creek’s *Hydrogenobacter sp*. population appeared to be so recombinogenic that it more closely resembled a sexual species than a clonally evolving microbe. Together, this work demonstrates that a randomized single-cell approach can be useful for the exploration of previously uncultivated microbes from community composition to population structure.

## Introduction

Characterization of microbial communities using cultivation independent high-throughput sequencing has revolutionized our understanding of microbial diversity [1] and function [2–4]. Sequencing of marker genes, mainly the 16S rRNA gene, has radically advanced our understanding of taxonomic diversity [5–7], while shotgun metagenomics provides a complementary snapshot of predicted functional diversity within microbial communities [4, 8–10]. Moreover, the last several years have seen dramatic improvements in metagenomic assembly and binning algorithms, leading to large-scale studies of metagenome-assembled genomes (MAGs) [11–17], which in some cases have identified “taxonomic blind spots” (i.e., lineages where taxa in amplicon studies have been missed due to primer bias [18, 19] and/or large 16S rRNA gene introns [20, 21]). While accurate assembly and quality control of MAGs remains a challenge, increasing confidence in MAG quality has been achieved over the last few years as tetranucleotide frequency (TNF) combined with differential coverage data are now producing high-quality MAG datasets [22, 23]. However, challenges associated with the analysis of strain level heterogeneity remain, as high levels of within-species heterogeneity can increase fragmentation of metagenomic assemblies, and contamination and redundancy of MAGs [24, 25], leading to the production of chimeric MAGs (e.g., the incorrect grouping of sequences from closely related strains) [26]. Alternatively, single-cell isolation, whole genome amplification (WGA), and shotgun sequencing enables access to the taxonomic and functional potential of microbial communities, albeit with some distinct advantages and disadvantages. For example, compared to bulk metagenomes, single-cell genomes provide more manageable genome assemblies [27], they enable the direct linkage between mobile genetic elements (MGEs) and their hosts [28–30], and provide data that are amenable to population genomics analyses as each single amplified genome (SAG) represents the genomic content of the individual, not a population. Naturally, single-cell sequencing comes with its own set of challenges as preparation remains technically challenging and labor intensive [31], and the reliance on WGA techniques such as multiple displacement amplification (MDA) can lead to sub-optimal genome quality due to stochastic amplification biases [32], complicating downstream analyses.

Despite the challenges associated with single-cell sequencing, when SAGs are of sufficient quality [33], single-cell genomics can reasonably substitute for isolate genomes in the analysis of natural microbial populations. For example, sets of closely related SAGs have previously been used for the calculation of recombination rates within *SAR11* consortia [34], the identification and characterization of hundreds of *Prochlorococcus* genomes [35], and for the deposition of thousands of untargeted SAGs spanning 28 marine samples to serve as reference genomes in the interpretation of meta-omics datasets [36]. In addition to the creation of reference genomes of uncultured microbial lineages, application of an untargeted single-cell genomics approach (i.e., not a priori targeting of the sampled cell populations) can help resolve questions related to the population structure and evolution of closely related microorganisms plucked directly from their natural habitats. The analysis of populations using single-cell approaches can further our understanding of microbial niche selection and the maintenance of population diversity. Population heterogeneity has recently been explored using metagenomic datasets in the form of strain tracking [37–41]. However the detection of genomic linkage and estimates of population-wide recombination rates across MAGs remains limited [42] (i.e., linkage can be determined only within the insert length of sequenced DNA inserts, usually ~ 300 bp [43]), but given that single-cell genomes represent individual cells, not populations, estimates of linkage and the subsequent evaluation of microbial recombination becomes more tractable.

Here, we evaluated the capacity of an untargeted single-cell genomic dataset (i.e., randomly sorted and whole genome amplified cells, with or without an amplifiable 16S rRNA gene) derived from a high temperature hot spring sediment sample to characterize the microbial community, from broad phylum level abundances and functional profiling to the within-species/population heterogeneity displayed by each of the most dominant lineages. To place this approach within the context of other contemporary technologies we compared our single-cell dataset of nearly 500 SAGs to paired amplicon and metagenomic datasets from the same sample. Specifically, we addressed the following three questions: (1) Are there biases associated with the taxonomic and functional gene profiles of the whole community when employing an untargeted single-cell approach? (2) Are there ecologically relevant gene content differences between MAGs and the corresponding set of same-species SAGs? And (3), Do dominant populations exist at different positions along a hypothetical evolutionary speciation gradient, i.e., from highly panmictic to structured? Taken together, this work demonstrates that an untargeted single-cell genomics approach can effectively characterize broad community structure of a low diversity sample while simultaneously providing a glimpse into the genomic heterogeneity of the dominant populations.

## Materials and methods

### Sample description

**Fig. 1:**
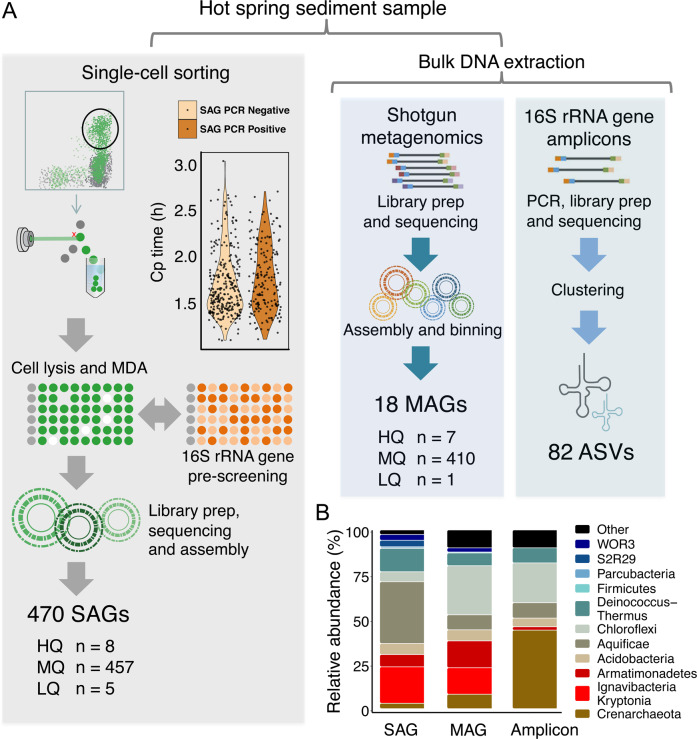
Production of paired sequence datasets and community composition. **A** Workflow for the generation of single amplified genomes (SAGs), metagenome-assembled genomes (MAGs) and 16S rRNA gene amplicons. In the single-cell sorting workflow, the crossing point (cp) value is indirectly proportional to the quantity of amplified DNA as a result of MDA amplification. No statistical difference was observed between 16S rRNA gene PCR positive and negative cp values (*p* > 0.05), suggesting that the 16S rRNA gene PCR is not a reliable indicator of the total quantity of DNA amplified during whole genome amplification. MAGs were generated from the bulk metagenome based on TNF and coverage profiles of the sediment metagenome, and the 16S rRNA gene amplicons were processed using a standard approach that involved the identification of amplicon sequence variants (ASVs) and classifying the resulting ASVs against the Silva database. **B** The community composition of this single sediment sample using the three approaches. Specifically, note the lack of Kryptonia and Armatimonadetes in the amplicon data, likely the result of primer mismatch.

A single hot spring sediment sample (pH = 8.0, 78 °C) was used to generate a 16S rRNA gene amplicon dataset, a bulk metagenome, and 470 single-cell genomes (Fig. 1A). This sample was collected from a Dewar Creek geothermal spring in Western Canada (49.9543667°, −116.5155000°) [44]. The sediment sample and the site itself have been described previously [18, 45].

### Preparation and sequencing of single amplified genomes (SAGs), bulk metagenome, and amplicons

SAGs were generated following the protocol outlined in Rinke et al. [46]. Briefly, single-cell isolation was performed using fluorescence-activated cell sorting (FACS) with 1X SYBR Green II to identify droplets containing DNA, representative of cells, which were arrayed into 384 well plates, followed by cell lysis and WGA using real time MDA. Within the FACS sort window, cells were randomly sorted without delineation of cell size or density, or any other optical properties. Each MDA product was then subject to a 16S rRNA gene amplification screen and scored as either positive for 16S rRNA gene amplification or negative based on the presence or absence of a PCR product. The primers used for the 16S rRNA gene PCR reactions were 926fw/1392r primers (see Trembley et al. for sequences) [47], targeting both archaeal and bacterial 16S rRNA genes. PCR conditions were performed according to DOE JGI standard protocols [46]. MDA-positive wells identified based on the analysis of qPCR amplification profiles and melt curves to assess reaction specificity. No significant difference was observed in crossing point values between MDA products with an amplifiable 16S rRNA gene versus those without (Fig. 1A). All single-cell MDA product wells that yielded MDA amplification curves above negative controls were passed to the JGI library production pipeline where libraries were prepared, followed by sequencing on the NextSeq platform (Illumina) where 75% of the libraries had read counts ranging from 7.5 to 35 million sequences. This resulted in 470 useable SAGs (Fig. 1A).

A bulk sample metagenome was constructed as described previously [18]. Library preparation was performed according to the protocol laid out in Bowers et al. [48] using the Nextera XT low biomass protocol without MDA amplification. The bulk metagenome library was sequenced on the HiSeq 2000 platform (Illumina), yielding 9.1 million reads.

Amplicon data were prepared by extracting DNA from 500 mg of sediment with the FastDNA® SPIN Kit for Soil (MP Biomedicals, Santa Ana, CA, USA), according to the manufacturer’s instructions. DNA was eluted using 50 µl DNase/Pyrogen-Free Water and stored at −20 °C. The primers used targeted the V6–V8 variable region of the SSU rRNA gene of bacteria, archaea, and eukaryotes. The library was quantified using the Qubit HS kit (Invitrogen, Carlsbad, CA, USA) and diluted to 4 nM. Amplicon libraries were prepared and sequenced using the MiSeq platform (Illumina). QIIME2 was used to analyze 16S rRNA gene sequence data [49]. Raw reads were quality controlled and denoised and sub-OTUs were formed using the deblur plugin [50]. Taxonomic assignment was performed with the feature-classifier plugin [51], and the classifier was trained on the Silva database, release 132 [52]. We elected to use the V6–V8 primers, as this primer set was used to identify taxonomic blind spot lineages from an earlier study at this site [18]. However, we acknowledge that all primer sets are biased to varying degrees, and as such a ‘blind spot lineage’ is only specific to the employed primer set.

### SAG and bulk metagenome assemblies and SAG/MAG quality control

All single-cell genome sequences and the bulk metagenome were quality checked and screened in the same manner. Reads were assembled using SPAdes (version 3.6.2) [53], and metagenome binning was performed using MetaBAT with default parameters. MetaBAT uses composition and coverage information to create the metagenome-assembled genomes (MAGs), and applies a minimum contig size of 2500 bp [54]. All MAGs and SAGs were quality assessed using CheckM [26], and the quality of each genome is reported in Supplementary Data Table including categorization into MISAG/MIMAG standards [33]. Genes were called and annotated using the integrated microbial genomes (IMG) system at the DOE Joint Genome Institute [55]. Furthermore, all genomes were subject to a combined pairwise genomic ANI analysis using fastANI [56] to define species-level clusters. Finally, 16S rRNA genes were extracted from SAG/MAG assemblies and used in primer mismatch analyses. See Supplementary Materials for additional details on bin splitting, pairwise genomic ANI clustering, and SSU rRNA collection and primer mismatch analyses.

### Metabolic profiles

To screen all Dewar Creek genomes (SAGs and MAGs) for their metabolic attributes, a set of 121 HMM profiles of specific metabolic markers were used to search each genome. These HMM profiles were obtained from a previous study published by Anantharaman et al. [13]. We also cross-reference these results with the functional annotations provided by the IMG annotation pipeline [57].

### Concatenated marker gene phylogenies and 16S rRNA gene phylogeny

Since hot springs often consist of rapidly evolving microorganisms [58], sometimes prone to horizontal gene transfer [59] and subsequently to variation in placement in single gene and/or concatenated multi-gene trees [60], we produced multiple trees including a 16S rRNA gene tree and concatenated marker gene trees using the following sets of markers: a set of 56 single copy conserved markers (UNI56) [61], a set of 16 ribosomal protein markers [62] and another tree using the concatenation of the three subunits of the RNA polymerase gene [63]. See Supplementary Material for additional details on alignments and tree construction.

### Identification of mobile elements: viruses, plasmids, and CRISPR spacer predictions

MGE sequences including phage, prophage, and plasmids were identified in the SAG, MAG and bulk metagenome datasets, using VirSorter [64] for viruses (phage and prophage) and PlasFlow [65] for plasmids. Only contigs greater than 10 kb were counted as MGE hits. Beginning and ends of contigs were also screened for overlap to determine circularity using the compute_overlap function in Biopython [66], however, no circular MGEs were found. MGE diversity was assessed with TNF clustering using all MGE contigs greater than 10 kb as input, and processed using the oligoFrequency function within the BioStrings package in R. MGE TNF comparisons were performed using PERMANOVAs from the vegan package in R. CRISPR spacers were identified using the IMG/VR CRISPR spacer database [67]. Briefly, CRISPR elements were identified using the IMG annotation pipeline, which relies on the programs CRT [68] and PILER-CR [69] to identify CRISPR-Cas proteins, spacers, and repeat sequences.

### Delineation of the populations within each dominant species

In preparation for SNP calling, the most complete SAG with a contamination estimate below 5% from each of the 95% ANI groups was identified and used as the reference genome. Reads of all SAGs were mapped to the references and SNPs were called using the MIDAS pipeline [40]. Phylogenies of the dominant populations were reconstructed by identifying variant sites between groups of within-species SAGs, followed by the production of a neighbor joining tree. Recombination was assessed with SNP linkage disequilibrium (LD) profiles for the *Hydrogenobacter sp., Kryptonium sp*., and *Thermus antranikianii* lineages. See Supplementary Material for additional details on SNP calling, population specific phylogenies, and for the determination of relative recombination rates.

## Results and discussion

### A snapshot of microbial community diversity at Dewar Creek hot spring via amplicon, shotgun metagenomic and deep single-cell sequencing

Dewar Creek hot spring is a geothermal spring located in the Purcell Wilderness of British Columbia. It was the site of a prior study investigating the relationship between hot spring temperature and microbial community composition [70], and was the site for the discovery of the *Candidatus* Kryptonia, which had been previously missed from amplicon studies as a result of primer mismatches [18]. To expand on these studies, and to assess the capacity of single-cell sequencing for whole community reconstruction using an untargeted approach, we isolated random cells using FACS, and sequenced 470 SAGs from a single Dewar Creek hot spring sediment sample (pH 8.0, T = 78 °C). We then compared SAG-based community composition to MAG and amplicon datasets from the same sample (Fig. 1).

Since the sequenced SAGs were not targeted to any specific taxonomic group, we first determined which cells would have been missed, had the standard single-cell genomic workflow requiring a 16S rRNA gene based amplification prescreen [46] been applied prior to shotgun sequencing. This untargeted approach has been previously taken by Pachiadaki et al. [36], though not for the direct comparison of untargeted SAGs, MAGs, and amplicons. In total, 470 SAGs with sufficient MDA product were shotgun sequenced and 287 of these SAGs were negative for an amplifiable 16S rRNA gene during our 16S rRNA gene prescreen. This means that 287 single cells (61%) would have failed a 16S rRNA gene PCR quality control step and as a result, would not have been shotgun sequenced if the prescreen was required (Fig. 1A). Furthermore, after extracting 16S rRNA genes directly from the SAG assemblies, we found that 33% of the 16S rRNA gene sequences either had mismatches to the employed 16S rRNA gene primers or had large introns, both of which are known to hamper efficient amplification [18, 20, 21, 71].

**Fig. 2:**
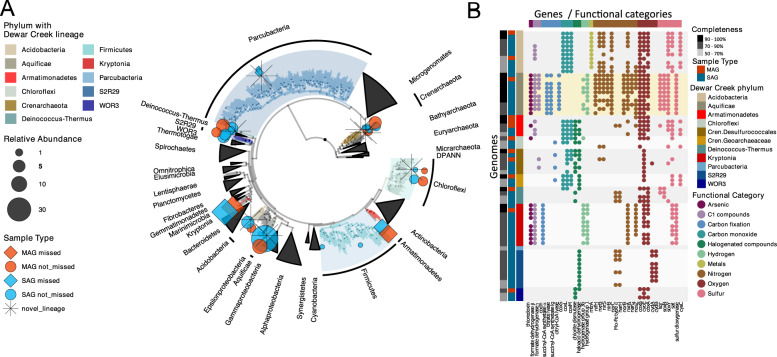
Phylogenetic composition and functional potential of the dominant Dewar Creek SAGs and MAGs. **A** Concatenated 56 marker gene tree that includes bacteria and archaea (rooted with the archaea). Dewar Creek genomes are denoted by colored symbols (circles and diamonds). A star next to a lineage indicates novelty (as determined by sharing less than 65% of identity in the *rpoB* gene to any sequence in the reference database) and a diamond denotes lineages for which more than 80% of the genomes within that lineage failed our 16S rRNA gene primer matching criteria. **B** Functional profile of the top 10 Dewar Creek SAGs and MAGs per lineage (<10 for many) classified at the phylum level. The functional profiles of each set of lineage-specific SAGs and MAGs are consistent, and the Aquificae, the phylum with the most diverse functional potential, is highlighted in yellow. Gene names are displayed as *x*-axis labels and these are grouped by functional categories.

Next, we found that each sequencing approach produced broadly similar profiles when considering the presence and absence of taxa (Fig. 1B and Supplementary Fig. 1). This low diversity community consisted of 12 major phyla including members of the Aquificae, *Candidatus* Kryptonia, Deinococcus-Thermus, Acidobacteria (or Gal08 Candidate phylum based on 16S rRNA gene sequences alone), Armatimonadetes, Chloroflexi, Firmicutes, Parcubacteria of the Patescibacteria/Candidate Phyla Radiation (CPR), Candidate phylum WOR3, Candidate phylum S2R29 (Calescamantes from GTDB-tk), and two potentially novel Crenarchaeota lineages (Fig. 2A, Supplementary Figs. 2–4, and Supplementary Data Table). This limited, yet largely understudied diversity was consistent with bulk metagenome read mapping, as 90% of reads could be mapped to the extracted MAGs, indicating that few lineages were left unbinned. While the overall taxonomic composition produced by each sequencing approach was broadly similar, differences did exist between the three datasets. Specifically, the *Candidatus* Kryptonia, Armatimonadetes, and Parcubacteria phyla were largely missing from the amplicon dataset, which had been previously identified as taxonomic blind spot lineages as a result of either primer mismatches (*Candidatus* Kryptonia—100% missed [18], and Armatimonadetes—92% missed) or large intergenic space within the 16S rRNA gene sequence (Parcubacteria—100% missed) (see Supplementary Material for mismatch criteria) [20]. Additional taxa missing from the amplicon dataset included the two candidate phyla S2R29 and WOR3, though mismatches or introns could not explain their absence.

The SAG and MAG datasets were similar taxonomically, although the Chloroflexi were underrepresented in the SAG dataset (5% of SAGs) as compared to both the MAG and amplicon datasets (22% and 27%, respectively). While an explanation for this discrepancy is not completely clear, we suspect that their possibly filamentous morphology may be connected to difficulties with sorting as noted previously [72]. The only phylum level lineage restricted to the SAG dataset was the S2R29 candidate phylum. We observed a relatively high proportion of S2R29 among SAGs (*n* = 17 SAGs, 4% of SAGs) and speculate that their relatively small genome size of ~1.5 Mb may suggest small physical size, and therefore, a greater likelihood to be sorted over other more morphologically complex microorganisms like the Chloroflexi, though we acknowledge that in general, genome-sizes are not consistently correlated with cell sizes [73]. Regardless of the physical reason so many S2R29 SAGs were successfully sorted, we now know that they are a member of the Dewar Creek microbial community, and as such, we looked for evidence of this phylum within the metagenome, even though MAGs assigned to this phylum were not produced. Following a simple read-recruitment exercise, we found that this phylum was present in the bulk metagenome, although at a very low level (<1% of the total metagenomic read set), likely too low to be binned effectively. This novel phylum level lineage will be explored in more detail in a future publication.

### Species diversity in Dewar Creek is largely limited to one species group per phylum

With the goal of applying population based analyses to the dominant species-level lineages, we next collapsed the SAGs and MAGs into 95% average nucleotide identity (ANI) groups, corresponding to the accepted operational species-level cutoff [56], in order to obtain specific sets of genomes to be used in downstream population analyses. Surprisingly, most observed phyla were constrained to a single 95% ANI group as 12 of the 21 ANI groups made up 95% of the genomic dataset (i.e., SAGs and MAGs). Moreover, the dominant ANI genome clusters were highly similar, with average ANI percentages over 97% (Supplementary Fig. 5), suggestive of sequence discrete populations [74, 75]. Therefore, in the absence of disturbance, cohesive forces are likely to maintain the *status quo*, i.e., a stable species composition [76]. However, this of course depends on the level of resolution, as additional samples would be required to further test this observation.

### Functional diversity suggests a range of metabolic strategies, from potential partnerships to the do-it-yourself microbes

The metabolic capabilities of the resident Dewar Creek hot spring microorganisms were assessed with a screen of 121 metabolically relevant hidden Markov models [13]. Most of the surveyed taxa contained pathway deficiencies (Fig. 2B), perhaps suggestive of metabolic partnerships [71], though we acknowledge that this may also be the result of incomplete genomes. In stark contrast however, the *Hydrogenobacter sp*. (phylum Aquificae) group of SAGs and MAGs displayed a wide range of functional potential, including the utilization of reduced forms of hydrogen and sulfur compounds as electron donors, and nitrate, elemental sulfur, and oxygen as electron acceptors [77], as well as marker genes for the reverse tricarboxylic acid cycle [78] which is thought to be one of the most ancestral forms of carbon fixation [79]. Given the high frequency of this *Hydrogenobacter sp*. lineage in the current dataset (34% of SAGs), its vast metabolic repertoire (Fig. 2B), and the recent acknowledgment that other Aquificales such as the *Sulfurihydrogenibium spp*. are the dominant primary producers in their respective habitats [78], we probed deeper into the within-species/population heterogeneity of this lineage alongside other dominant Dewar Creek species-level lineages including *Kryptonium sp*. (*Candidatus* Kryptonia) and *Thermus antranikianii* (phylum Deinococcus-Thermus).

### SAGs reveal genomic diversity extending beyond metagenomic bins

**Fig. 3:**
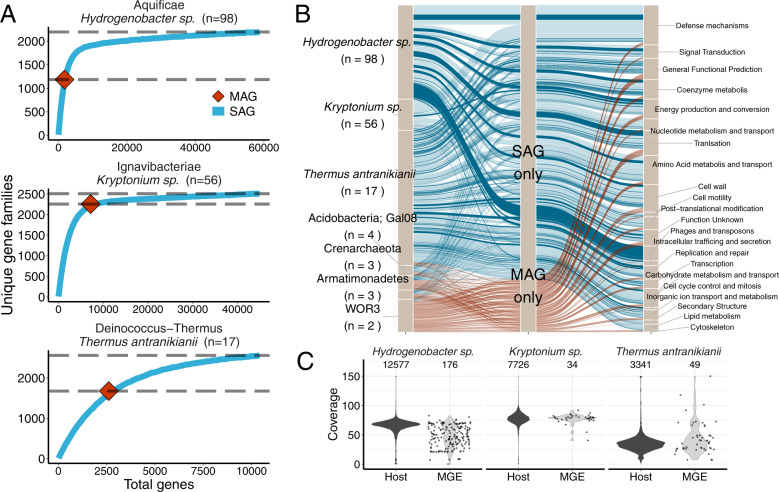
The collection of within-species sets of SAGs indicated that MAGs lacked a proportion of the accessory component of the Dewar Creek population genomes. **A** SAG rarefaction curves of the dominant lineages: *Hydrogenobacter sp*., *Kryptonium sp*., and *Thermus antranikianii* (blue lines) shown together with the total number of unique gene families within each corresponding MAG (red diamond). The difference between the two gray dotted lines indicates the number of unique gene families missed by the MAG. **B** Alluvial plots showing the gene families unique to SAGs and MAGs and their corresponding COG functional annotations (level 3) from all Dewar Creek lineages with paired SAGs and population MAG. The alluvial flows with darker coloring (SAG only subset) are those genes derived from contigs identified as MGEs. **C** Coverage of MGE genes within the bulk metagenome suggests variation in viral coverage as compared to the host genome. Coverage plots are based on read mapping of bulk metagenome reads to each of the SAG genomes. Note: Singletons from the unique gene family datasets have been removed from the SAG datasets in order to reduce the effect of potential contamination, i.e., the gene had to be observed in at least two SAGs.

Based on a previously published coarse-grained comparison, taxonomically related SAGs and MAGs have been shown to produce similar genomes [80]. However more recently, Nelson et al. [81] revealed evidence supporting the commonly held notion that since metagenomic binning usually relies on compositional signatures, that outlying genes are sometimes missed during the binning process including genes encoding ribosomal RNAs, transfer RNAs, MGEs and large numbers of functionally unknown genes. While both studies are instructive, neither performed a direct paired comparison of SAGs to a corresponding population MAG from the same sample. The Alneberg work compared SAGs to previously sampled and extracted MAGs from the Baltic Sea [80], while the Nelson study compared MAGs to corresponding NCBI RefSeq genomes [81], but to the best of our knowledge our current work is the first to compare SAGs to MAGs derived from the same environmental sample. After grouping the genes of our SAGs and MAGs into orthologous groups (i.e., gene families) and assigning them to COG annotation categories, we found that, even when excluding singletons, the collective SAG sets routinely captured more gene family diversity than the population MAG (Fig. 3A). However, this pattern was reversed when the ratio of SAGs to the single population MAG dropped below ~5, as the variation in SAG genome recovery combined with a small SAG sample size became more limiting (Fig. 3B and Supplementary Fig. 5a, b). Of the dominant lineages, very few unique gene families were exclusively found in the MAGs, and those that were observed cannot be ruled out as representing contaminating sequences as a result of mistakes during the assembly or binning processes. While we show nearly saturated gene family diversity within each dominant lineage, we must note that these curves would continue to rise had we retained singleton orthologues, thus producing a picture that is similar to the expected large pangenomes of most microorganisms [82]. Nevertheless, it is clear that the collective gene content obtained from each of the dominant SAG sets (i.e., *Hydrogenobacter sp., Kryptonium sp*., and *Thermus antranikianii*) (Fig. 3A) provides a more complete survey of the gene family diversity within a given species-level lineage or population. Moreover, the most consistently missing COG category from the MAGs was the phage and transposon category (Fig. 3B), which was likely missed due to variation in nucleotide composition and/or variation in coverage, as described in the next paragraph. Our results agree with the recent work of Nelson et al. [81], and another study that utilized a simulated low-complexity metagenome composed of taxa with high plasmid and genomic island content, demonstrating that MGEs were often missed during MAG reconstruction [83].

**Fig. 4:**
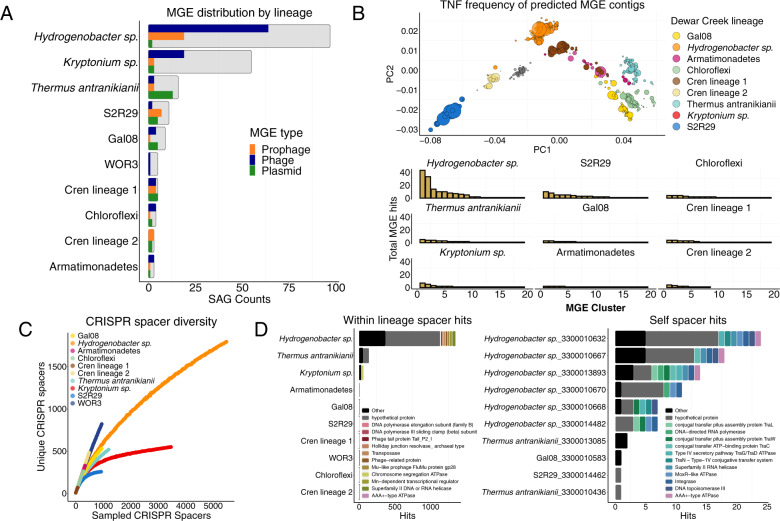
MGE abundance, diversity, and subsequent spacer diversity within Dewar Creek SAGs. **A** Presence/absence of each MGE type (phage, prophage, or plasmid) per SAG where the total number of analyzed SAGs are noted by the background, gray bar. Genomes considered positive for an MGE must have had at least 1 MGE contig greater than 10 kb. **B** MGE diversity. Tetranucleotide frequency of each MGE contig (excluding prophage as host genome cannot be excluded) (top). MGE counts per 99% ANI group (bottom) **C** Rarefaction curves noting spacer diversity within each lineage. **D** Spacer matches hitting another SAG within the same lineage excluding self hits (left), and self (within genome) hits (right).

To investigate the missing MAG MGE gene families in more detail, we screened all SAGs and MAGs for the presence of phage/prophage and plasmids using the MGE screening tools VirSorter [64] and PlasFlow [65], respectively. Of the three dominant lineages, the *Hydrogenobacter sp*. and *Thermus antranikianii* lineages had high MGE content (Fig. 4A), which were correspondingly the lineages with the largest increase in the accessory genome component in the SAG to MAG comparisons (Fig. 3A). Furthermore, we found that the abundance, as a result of bulk metagenome read mapping to the SAG contigs flagged as MGEs, was much more variable than the non-MGE contigs (Fig. 3C), reflective of mobile element biology, as higher than average read coverage might suggest MGE replication [28], and/or sporadic coverage could indicate an uneven distribution of MGEs within a given host population, such as that observed with the *Hydrogenobacter sp*. population (Fig. 3C).

### Population specific MGEs reflect the extent of lineage-specific CRISPR-Cas immunity

Of the 470 SAGs, 70% had at least a single predicted MGE sequence greater than 10 kb in length (phage, prophage or plasmid), and of all identified Dewar Creek lineages, only one, *Candidatus* WOR3, was free of predicted MGEs (Fig. 4A). The identified MGEs were lineage-specific (Fig. 4B, PERMANOVA *p* < 0.05) which is consistent with earlier work [84], and their abundance tracked with a correspondingly high percentage of SAGs with at least one CRISPR array. (Ninety percent of all SAGs contained at least one CRISPR array.) The increase in the prevalence of CRISPR-Cas immunity against the MGE gene pool has been previously related to a cost-benefit scheme where in mesophilic communities, viral mutation rates exceed thresholds beyond CRISPR-Cas’s ability to provide sufficient immunity at which point CRISPR-Cas systems are purged from the host [85]. However, in high temperature environments lower overall mutation rates are typically observed [86], thus lowering the cost of maintaining high levels of CRISPR-Cas in thermophilic microorganisms [85]. While we did not compare our dataset directly to similar mesophilic communities, our results do follow the logic presented in Weinberger et al. [85] as most of our sampled populations contained extraordinarily high spacer diversity (Fig. 4C). Furthermore, those lineages with very high spacer diversity corresponded to a similarly large population specific MGE gene pool, as demonstrated by the *Hydrogenobacter sp*. lineage in Fig. 4B. The *Hydrogenobacter sp*. MGEs exhibited the clearest examples of plasmid-like elements and phage sequences, including putative ICE (Integrative Conjugative Element) sequences where two of the syntenic ICE-like sequences contain additional chromosomal sequence and all five of the contigs contain the core Type IV secretion system machinery (Supplementary Fig. 6a) which are typically associated with ICE plasmids [87]. Our analysis also reveals the genomic context of phage integration for a specific *Hydrogenobacter sp*. phage. The phage itself contains several phage structural genes, an integrase, and the terminal inverted repeats indicative of a possible integration site (Supplementary Fig. 6b).

Since the abundance and diversity of spacer elements appears to be directly connected to the circulating species-specific MGE pools, we next identified spacer targets within the 470 SAG dataset in order to detect whether targets span different lineages, are constrained to the same lineage and/or are observed on self-genomes (i.e., self-hitting spacers). In agreement with previous work [84, 88], there were effectively zero cross-lineage spacer hits, which might be expected given the host-specificity of the MGE pools (Fig. 4B). However, high levels of within lineage spacer/target matches were observed alongside a number of self-targeting spacers (Fig. 4D). Interestingly, there appears to be a high degree of crosstalk, specifically within the *Hydrogenobacter sp*. population, where the spacers of one SAG target an MGE of another SAG within the same population (Fig. 4D, left panel). Correspondingly, the *Hydrogenobacter sp*. population also contained the highest level of self-targeting spacers (Fig. 4D, right panel). Though self-targeting spacers are typically deemed detrimental to a cell’s fitness (e.g., autoimmunity is usually harmful [89]), self-targeting spacers have recently been shown to be quite common as they are observed in one fifth of all CRISPR-harboring bacteria [90]. Furthermore, they may provide some benefit as self-targeting spacers have been associated with the prevention of prophage induction [91], or in other cases, they may expedite the removal of prophage sequence [92].

### Population heterogeneity at Dewar Creek: from the highly panmictic to the more structured populations

**Fig. 5:**
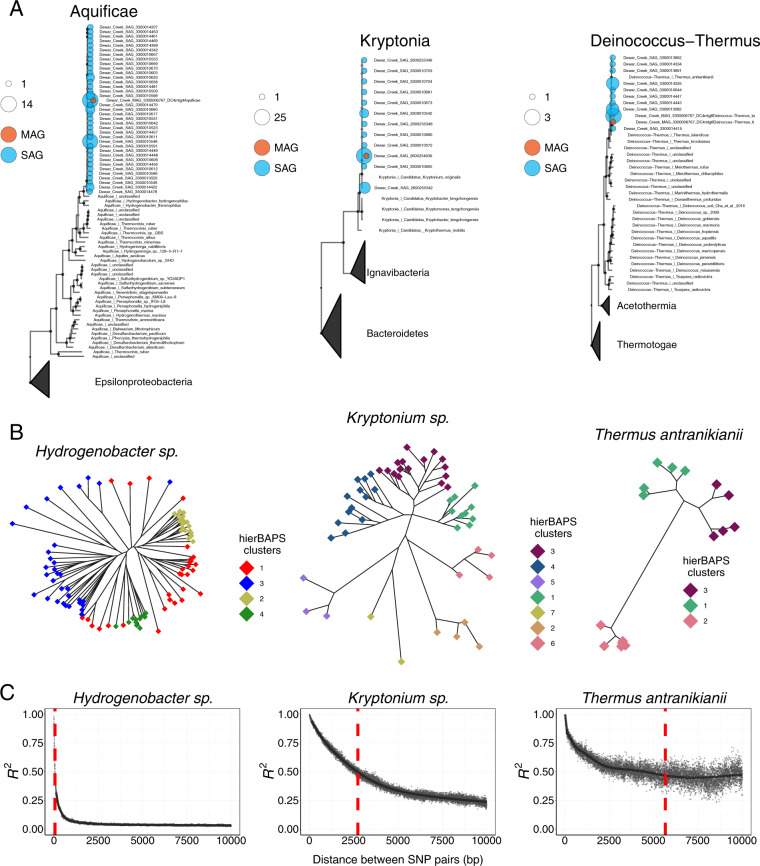
Dominant Dewar Creek populations belonging to the Aquificae, Kryptonia, and Deinococcus-Thermus phyla. **A** Lineage-specific conserved marker trees (same 56 markers used in above bacteria/archaea tree), where all available marker sequences were used for each phylum; sequences were depreplicated based on RNA Polymerase clustering at 100, 100, and 90% similarity for the Aquificae, Kryptonia, and Deinococcus-Thermus sequences, respectively. Dereplication was performed to remove redundancy and generate a representative set of marker sequences for each phylum. Dewar Creek genomes were also dereplicated, but at 100% RNA Polymerase identity where larger bubbles indicate an increase in the number of genomes for a given RNA Polymerase gene cluster. **B** SNP trees where variant positions were identified based on whole genome alignments. Tip colors correspond to hierBAPs Bayesian clustering results. A permanova analysis was performed to compare tree topologies with hierBAPs clustering, identifying inconsistences within *Hydrogenobacter* sp. (*p* < 0.05), but not *Kryptonia sp*. or *Thermus antranikianii* species (*p* > 0.05). **C** SNP linkage disequilibrium (LD) curves demonstrating evidence for more recent recombination within the *Hydrogenobacter sp*. The red dotted line represents the distance where the LD curve crosses an *R*^2^ threshold of 0.5, i.e., the distance in base pairs where 50% of the SNP pairs are no longer correlated. A smaller distance at an *R*^2^ of 0.5 indicates a higher rate of recombination.

This high-depth, untargeted, single-cell sampling of one low diversity sediment sample resulted in a high number of SAGs from three distinct species-level lineages (95% ANI groups) most closely related to *Hydrogenobacter sp*. (*n* = 98)*, Kryptonium sp*. (*n* = 56), and *Thermus antranikianii* (*n* = 17). RNA polymerase beta-subunit clustering showed that the genomes from each of these populations differed from available reference genomes. While same-population SAGs were highly similar, they were not identical, as a number of unique RNA polymerase beta-subunit OTUs were detected (Fig. 5A). To probe deeper into the heterogeneity within each population, we assessed population diversity based on the identification of single nucleotide polymorphisms (SNPs) and estimated within-population recombination rates based on the assessment of LD profiles resulting from the pairwise correlation of all SNP pairs. Overall, we demonstrate that while each of these populations has a similar average genome-wide ANI, each population has unique structure, as the relative levels of recombination varied. This structure appears to coincide with the ability of each population to differentiate into distinct sub-species clusters (i.e., *ecotypes*) [93]. First, we show that the genetic structure of the three populations ranges from more clonal to highly panmictic, as variant based phylogenies combined with Bayesian Analysis of Population Structure (BAPS) [94, 95] show distinct sub-species clustering within the *Kryptonium sp*. and *Thermus antranikianii* populations; while the star-like phylogeny, discordance in BAPS clustering, and slightly elevated nucleotide diversity of the *Hydrogenobacter sp*. population (Supplementary Table 1) are suggestive of a quasi-sexual population, similar to a recently described thermophilic cyanobacterial population from Yellowstone [96]. These differences are further illustrated by the variation in LD profiles exhibited by each lineage (Fig. 5C). Briefly, LD curves can show the extent of SNP linkage (non-independence of alleles) spanning a reference genome where a strictly clonal population would exhibit no linkage while recombining populations exhibit various degrees of decay in linkage based on the extent of population-wide recombination. Based on our present analysis, it is clear that all three surveyed populations show LD profiles indicative of recombination (Fig. 5C). However, the overall extent appears to mimic the population structure that we observe in the variant site phylogenies (Fig. 5B), as the distance at which 50% of all SNP pairs becomes unlinked within the *Hydrogenobacter sp*. is only 100 bp whereas the 50% unlinked distance is 3000 and 4000 bp within the *Kryptonium sp*. and *Thermus antranikianii* populations, respectively (Fig. 5C). This relative rate of recombination within the *Hydrogenobacter sp*. lineage appears to be particularly high, but on par with some of the clinically relevant highly recombining bacteria including *Helicobacter pylori* [97] and *Neisseria meningitis* [98]. Furthermore, a recent analysis of SNP splits shows that very few microbial populations exhibit strictly clonal evolution and that the more rapidly recombining species such as *Helicobacter pylori*, recombine so often that they appear to be freely recombining, exhibiting quasi-sexual population structure [99], which may explain the very rapid decay in linkage within the set of *Hydrogenobacter sp*. SAGs.

While applying mathematical models to explain the variation in population structure is beyond the scope of our current analysis and underlying dataset (our current dataset is not optimal for defining true barriers to recombination as this would require additional sampling across ecological gradients); we nevertheless show that an untargeted single-cell dataset produced directly from an environmental sample without the biases associated with cultivation can begin to unravel some of the complexities associated with microbial selection and diversification. Specifically, our analyses connect the abundance and diversity of MGEs to variation in the levels of population-wide recombination. For example, the *Hydrogenobacter sp*. exhibit genome characteristics that resemble the *guns-for-hire* paradigm where components of defense systems such as site-specific nucleases reside on genomic islands (i.e., conjugative plasmids, a.k.a. ICE’s) that are prone to HGT [100], and as such, defense and MGE genes display a higher mutational burden than genes related to informational processes as noted in Iranzo et al. [101] and observed here (Supplementary Fig. 6).

## Conclusions

Our current work demonstrates the utility of an untargeted single-cell sorting and sequencing approach for the analysis of community-wide taxonomic and functional profiling, that in specific circumstances, i.e., when overall species diversity is low, has the capacity to more comprehensively dissect the heterogeneity within dominant community members. We found that when species diversity is low, such as within our Dewar Creek sediment sample, few differences were observed in broad phylum level taxonomic profiles produced by the three separate sequencing approaches: an untargeted set of nearly 500 SAGs, a bulk metagenome with corresponding MAGs, and a paired amplicon dataset. Furthermore, the differences that were observed only aided to expand known diversity as *blind-spot* lineages agreed with previous metagenome surveys [18, 20] and an additional lineage, candidate phylum S2R29, was only observed in the SAG dataset but missing from the MAG and amplicon datasets, appearing to be a member of the rare-biosphere based on bulk metagenome read mapping to the S2R29 SAGs. Given that we had paired SAGs and MAGs, we further explored the differences between these two types of uncultivated genomes, which led us to the identification of mobile element gene pools that went largely missing from the MAGs. Furthermore, we found that population specific MGE content reflected the diversity of the resident CRISPR spacers. Finally, since we had a sufficient number of SAGs corresponding to three dominant Dewar Creek lineages, we explored the variation in within-population heterogeneity noting that each population exhibited a footprint of recombination, though the *Hydrogenobacter sp*. population appeared to be so recombinogenic that it more closely resembled a sexual species than a clonally evolving one.

This work demonstrates that single-cell sequencing has great potential for the characterization of whole microbial communities while simultaneously offering a glimpse into the genome evolution of dominant populations. We hope that this study can be viewed as a preview of the resolving power that single-cell sequencing can afford, especially as larger multi-sample single-cell sequencing studies are undertaken. As we continue to explore the genomic heterogeneity of uncultivated microbial populations that dominate Earth’s ecosystems, we may begin to unravel some of the important questions in microbial ecology and evolutionary biology.

## Supplementary information

Supplementary Material

Supplementary Data Table

## Data Availability

All final genome data for this work can be found on the IMG website (https://img.jgi.doe.gov/) [55]. Genomes and metagenomes used in the current study can be found using the IMG taxon IDs shown in the Supplementary Data Table, which also includes genome quality, genome size, tRNA count, and sample type columns. The amplicon dataset has been deposited in SRA under the accession number SRR17022153.
