# Supplementary material for: Dissecting the dominant hot spring microbial populations based on community-wide sampling at single-cell genomic resolution
Source: ISME J. 2021 Dec 30;16(5):1337–47. doi: 10.1038/s41396-021-01178-4 (PMC9039060; doi:10.1038/s41396-021-01178-4)
Supplement: Supplementary file 1 — Supplementary Material [file 41396_2021_1178_MOESM1_ESM.pdf]

Materials and Methods Supplement

Bin splitting, and additional genome quality assessment

Two MAGs that classified as members of the Acidobacteria and Deinococcus-Thermus phyla had high contamination estimates of 74% and 68%, respectively. After analyzing each MAG in greater detail using both refineM [1] alongside manual TNF frequency and coverage plots (Supplementary Figure 2a), we found that each could be split to create two additional MAGs. The Acidobacteria MAG could be separated using read coverage and TNF into a low coverage and high coverage MAG, after which the contamination was reduced to 5.8% and 3%. The contaminated Deinococcus-Thermus MAG appeared to represent two distinct species, as the refineM BLAST taxonomy assignments were largely split between *Thermus antranikianii* and *Thermus islandicus,* corresponding to two different clouds on the TNF plot (Supplementary Figure 2b). Separation of these sequences reduced the contamination estimates to less than 5% for the two new MAGs. Furthermore, the TNF-based diversity of the *Thermus antranikianii* MAG largely coincided with the diversity of the dominant Deinococcus-Thermus SAG population (Supplementary Figure 2c), thus the *Thermus antranikianii* MAG was used for the downstream Deinococcus-Thermus SAG to MAG comparisons.

SSU rRNA collection and primer mismatch analysis

16S rRNA genes were extracted from the assemblies using cmsearch of the Infernal package [2] and the RF00177 covariance module of the 16S rRNA gene [3]. Only 16S rRNA genes greater

than 1 kb in length were used in subsequent analyses (n = 151 16S rRNA gene sequences). As expected, lower quality genomes were less likely to contain a 16S gene (completeness estimate average was 54% for genomes with a 16S gene, and 33% for those without). Once 16S rRNA genes had been collected and filtered to 1 kb and above, primer binding was assessed with PrimerProspector [4] using default parameters. Any sequence with an overall weighted score greater than 1.0 suggested that the 16S rRNA gene would be missed by the tested primer set (see Eloe-Fadrosh et al. 2016 for additional details [5]).

Construction of concatenated marker gene phylogenies and 16S rRNA gene phylogeny

Concatenated marker gene phylogenies were constructed by combining a dereplicated reference set of genomes together with query genomes (e.g. Dewar Creek SAGs and MAGs). Marker proteins were extracted from each genome using hmmsearch (version 3.1b2) and alignments were constructed with MAFFT [6] using the mafft-linsi option. Alignments were trimmed with trimAl 1.4 [7], removing sites when more than 90% of taxa contained a gap. For the 16 ribosomal protein tree and 56 marker gene tree, genomes were removed if they contained less than 50% of the markers in the set. The presence of all 3 subunits for the RNA polymerase gene were required for a genome to be included in the 3 subunit RNA polymerase phylogeny. Individual protein alignments were then concatenated to produce an alignment of 51,239 sites. Maximum likelihood phylogenies were constructed with IQ-TREE [8], using the WAG substitution model and 1,000 bootstraps. The set of reference genomes was collected by dereplicating the full set of IMG (Integrated Microbial Genomes) isolate genomes (64,005

genomes) [9] based on cd-hit [10] clustering of the RNA polymerase gene (*rpoB*) at 65%. This produced a dereplicated set of unique family-level genomes, spanning all bacteria and archaea (n=681).

Lineage-specific trees (Figure 5a and Supplemental Figure 4) were constructed in a similar manner, however, only the UNI56 marker set was used. Outgroups were selected as the nearest neighbor taxa from the full UNI56 archaea/bacteria tree. For these trees, the full set of genomes were collected for each phylum from IMG/M (Integrated Microbial Genomes / Metagenomes) [11], then dereplicated using the *rpoB* gene at different clustering levels, ranging from 90 to 100%. The clustering level was varied by clade in order to produce roughly 50 references per phylum. The reference set for the Crenarchaeota was dereplicated at 80% *rpoB* similarity, as this was a broader phylogenetic clade than the other sets of lineage specific trees. The query genomes (Dewar creek SAGs and MAGs) were dereplicated at 100% RNA Polymerase beta-subunit gene identity. The lineage-specific trees were constructed in the same manner as outlined above.

The reference set for the 16S rRNA gene phylogeny was based on sequences extracted from the 681 reference genomes used in the multi-marker gene trees. Dewar Creek SAG and MAG query sequences only included 16S rRNA gene sequences that were greater than 1kb in length (n = 151). The combined set of query and reference 16S rRNA genes was aligned using cmalign using the -matchonly option, resulting in an alignment length of 1534 bp, and the tree was

constructed with IQ-TREE [8] under the general time-reversible evolutionary model with 1,000 bootstraps. All trees (16S rRNA gene and protein markers) were visualized with ggtree [12] in R.

Relative abundance comparison between amplicon, SAG and MAG datasets

Since community composition was compared across SAGs, MAGs and amplicon datasets, the generation of abundance profiles from the three distinct approaches should be briefly described. Relative abundances of amplicon groups were the result of 97% OTU clustering, taxonomic assignment, and grouping at the phylum level. The SAG abundances were straightforward, as taxon assignments were based on the UNI56 maker gene tree, then counts were based on phylum level assignments. Taxonomy assignments of MAGs were also based on the UNI56 marker gene tree and abundances were based on read mapping where reads from the bulk metagenome were mapped to the collective set of MAGs using bbsplit from the bbtools package [13], where a read could only be mapped once.

Note on phylum level classifications

Candidatus Kryptonia is described as a phylum within NCBI and the corresponding publication [5] while the GTDB-Tk [14] places Kryptonia within the Bacteroidetes phylum, and *Candidatus* Parcubacteria [15] within the Patescibacteria phylum. For the current work, we are using the names from the original publications as both classifications are based on the commonly cited 16S rRNA phylum designations [16] and a concatenated ribosomal tree in the case of the

Patescibacteria [15], though we acknowledge the new names in GTDB-tk and note that specific phylum names are not of critical importance to this manuscript as much of the study focusses on comparisons between SAGs and paired MAGs, and further dissection within and between dominant populations.

Pairwise average nucleotide identity (ANI) and definition of species level clusters used in downstream analyses

Pairwise genomic ANI analysis was performed with fastANI [17]. Genome pairs were filtered to include only those pairs with an alignment fraction $\geq$ 70%, which were then grouped into clusters sharing $\geq$ 95% ANI using mcl [18]. Pairwise ANIs $\geq$ 95% were used to define species level clusters [19]. These ANI clusters were used for downstream intra-species analyses including gene family/orthologue clustering and population analyses.

Gene annotations, gene content comparisons and gene family diversity assessment

Genes were called and annotated using the Integrated Microbial Genomes (IMG) [11]. The naming of contigs and genes followed JGI's in house nomenclature and can be cross-referenced with the IMG webserver (img.jgi.doe.gov). For gene content comparisons, annotations of individual genes were used in combination with gene family clustering using OrthoFinder 2.1.3 [20], and the ANI/species level genome collections as input. In addition to clustering genomes into 95% ANI groups, completion cutoffs of 40% were used for ortholog clustering.

Identification of SNPs

In preparation for SNP calling, the highest quality SAG (SAG with the highest completeness estimate and contamination less than 5%) from each 95% ANI group was identified and used as the reference genome. Reads of all SAGs were mapped to the references and SNPs were called using the MIDAS pipeline [21]. Briefly SNP calling was done by mapping all reads belonging to genomes within an ANI group using bowtie2 (--very-sensitive, global alignment mode) and reads with less than 95% similarity to the reference, average read quality of less than 30, mapping quality less than 20, and base quality scores of less than 30 were discarded. For a SNP to be counted, it had to have a minor allele frequency (MAF) of at least 10%.

Whole genome phylogenies of the dominant populations

Population level phylogenies based on the variant sites between SAGs were created by taking a collection of within species genomes, identifying variant sites using NUCmer from the MUMmer package [22], then producing a neighbor joining tree. Identification of strain level clusters was performed via RhierBAPS [23] using the same NUCmer whole genome multiple sequence alignment as input, by partitioning each genome sequence into the appropriate cluster based on the allele frequencies within each cluster.

Estimates of recombination

We generated SNP linkage disequilibrium (LD) profiles for the *Hydrogenobacter sp., Kryptonium sp.*, and *Thermus antranikianii* lineages, where the MIDAS constructed SNP depth and frequency tables were used as input and converted to a SNP pair correlation matrix (R^2). LD plots were created by reading in the SNP pair correlation matrix, creating a table of R^2 values by distance, then plotting in ggplot2 [24]. The number of SNPs per kb was also calculated on a per gene basis and mapped to their corresponding annotations (COG database used in figures). SNPs per kb were calculated using the MIDAS script SNP_diversity.py (https://github.com/snayfach/MIDAS) [21].

Supplementary Figures and Tables

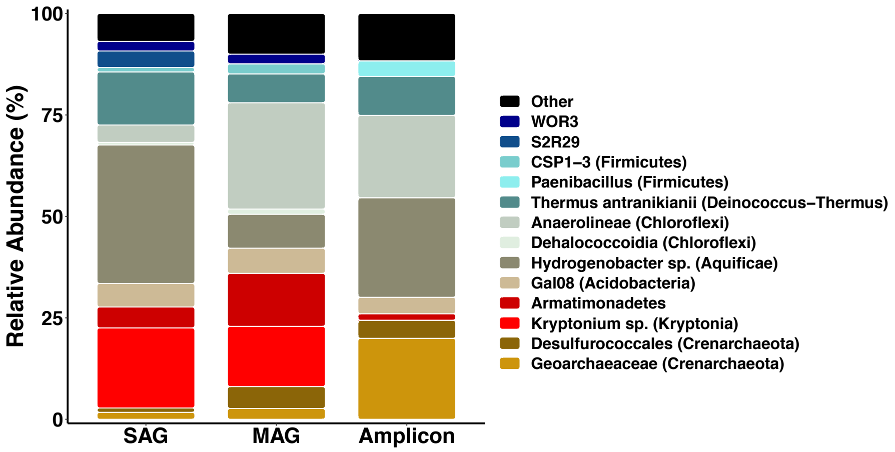

Supplemental Figure 1. The community composition of the single Dewar Creek sediment sample using the three approaches. This figure is similar to Figure 1b, but with more resolved taxonomic assignments.

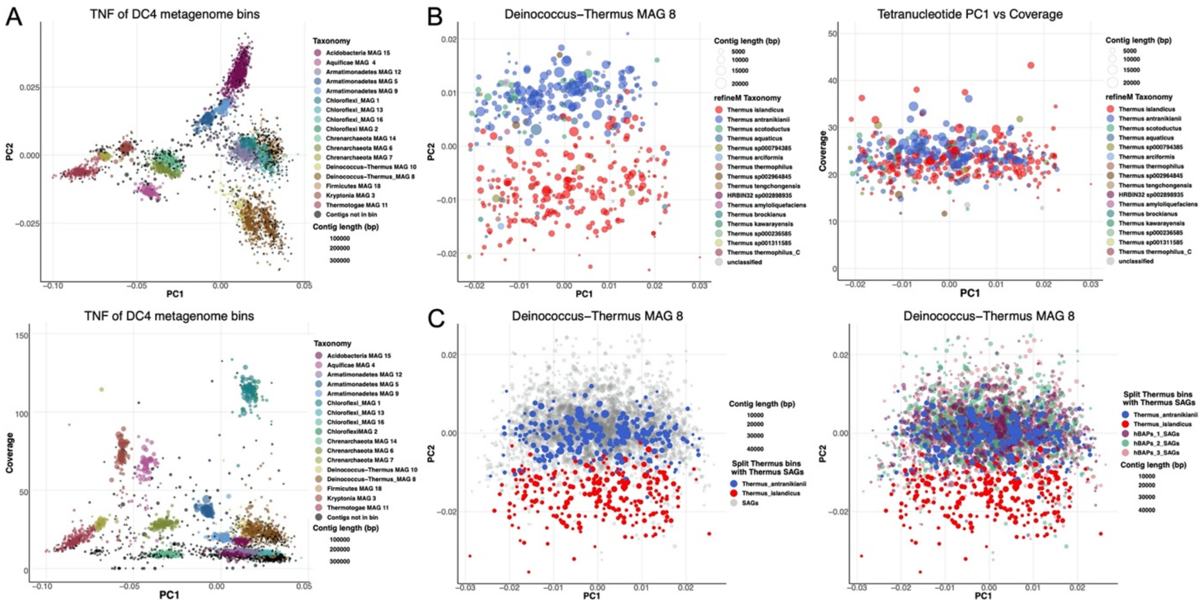

Supplemental Figure 2. Example of bulk metagenome bin cleaning. **a** Tetranucleotide frequency plot of all bins extracted from the bulk metagenome. All but one of the bins were either high or medium quality MAGs based on the MIMAG/MISAG standards [25]. **b** Demonstration of bin cleaning using the original highly contaminated Deinococcus-Thermus MAG 8, colored by contig taxonomy assignments. The majority of contigs classified to either *Thermus antranikianii* or *Thermus islandicus*. **b, left** shows two clear TNF compositional clouds and **b, right** shows that these bins could not be separated based on coverage and PC1 alone. **c** demonstrates that most Deinococcus-Thermus SAGs are most similar to the *Thermus antranikianii* MAG. **Left** MAG contigs combined with SAG contigs. **Right** shows the same plot, but where SAGs are colored by the HBAPS population clusters assigned to the *Thermus antranikianii* SAGs.

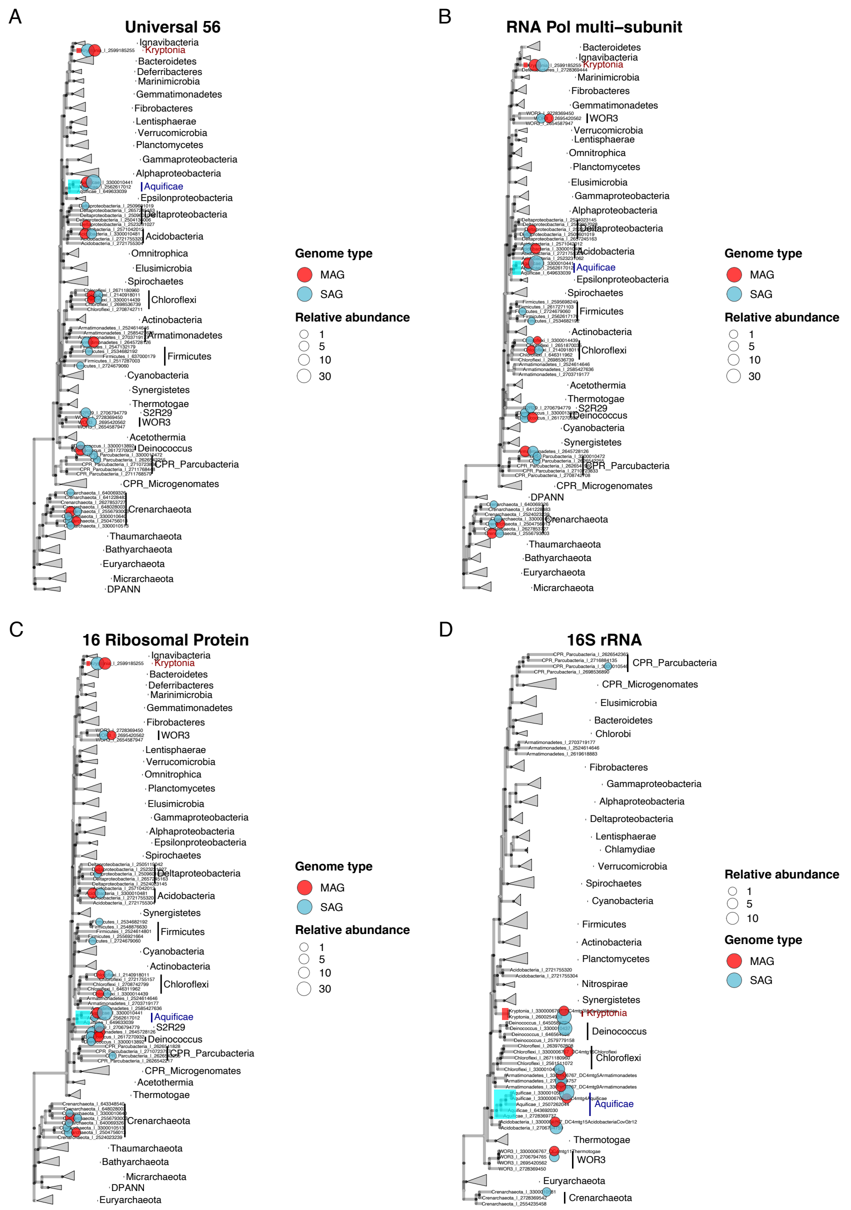

Supplementary Figure 3. Phylogenetic trees of the Dewar Creek bacteria and archaea using different phylogenetic markers and marker sets including **a** the 56 universal markers used in main manuscript figure (Figure 2a), **b** the three subunits of the RNA Polymerase gene, **c** a set of 16 conserved ribosomal proteins, and **d** the 16S rRNA genes derived from the same set of genomes used in the multi-protein phylogenies. Abundance counts within concatenated protein phylogenies (**a – c**) represent relative proportions within SAG and MAG datasets where MAG relative abundances are the result of bulk metagenome reads mapped to each MAG. Abundance counts within the 16S rRNA gene phylogeny are the result of 16S clustering at 87.5 % similarity (family level based on Yarza 16S rRNA standards [16]). Note: if any Dewar Creek lineages are missing from one phylum within one marker set, but present when using a different marker set, this means the genome either did not contain the markers or did not have enough markers to remain in the tree after quality filtering (cutoffs for the universal 56 marker genes and 16 ribosomal proteins were set to contain at least 50% of the marker set).

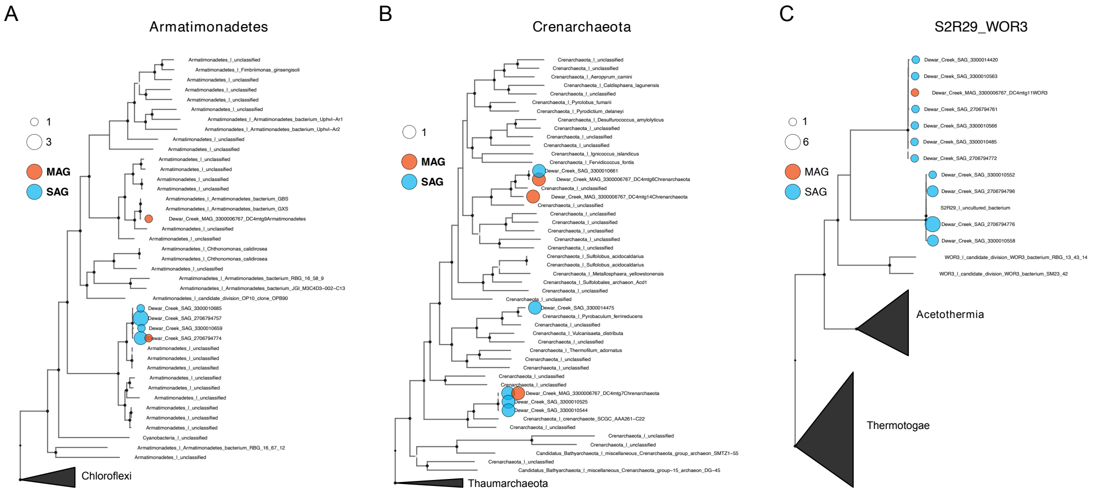

Supplemental Figure 4. Genome phylogenies of the abundant taxa excluding the Aquificae, *Candidatus* Kryptonia, and Deinococcus-Thermus phylogenies as these were shown in Figure 5a. Members of the **a** Armatimonadetes, **b** Crenarchaeota, and **c** S2R29 and WOR3 candidate phyla are displayed. The sizes of the Dewar Creek genome bubbles are based on 100% *rpoB* clustering. For example, when a bubble is larger than a value of 1, this means there are two or more identical genomes in that cluster based on the *rpoB* gene.

A

95% ANI group	Genomes	ANI%	SAG Only	MAG Only
Aquificae; *Hydrogenobacter sp.*	98	99	635	21
Ignavibacteriae; *Kryptonium sp.*	56	99	467	37
Deinococcus–Thermus; *Thermus antranikianii*	17	97	1168	89
S2R29; Unclassified	10	99	1740	NA
Acidobacteria; Gal08	4	99	235	120
Armatimonadetes; Unclassified	4	99	397	179
Crenarchaeota; Unclassified	3	99	340	379
WOR3; Unclassified	3	98	192	653

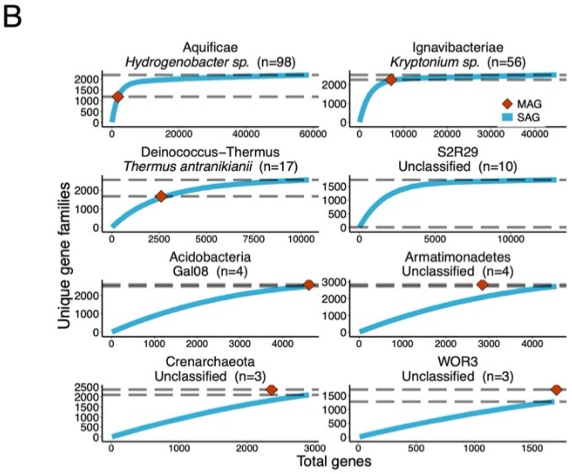

Supplementary Figure 5. a Table noting the average nucleotide identity (ANI) and the number of unique gene families observed in the SAG only or MAG only genomic subsets for each lineage. **b** Rarefaction curves highlighting the gene family diversity for SAGs (blue curves) and MAGs (red diamond).

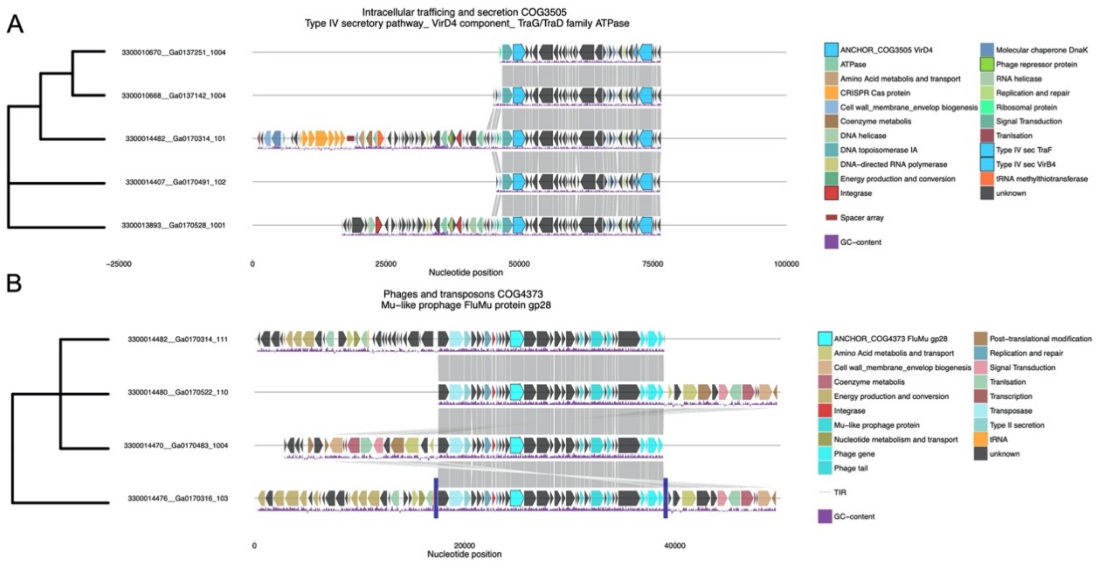

Supplementary Figure 6. a Observed synteny between 5 putative ICE plasmid sequences identified from two *Hydrogenobacter sp.* genomes. The "anchor" gene codes for a TraG/TraD ATPase, involved in T4SS transport. Sequences, 3300014482__Ga0170314_101 and 3300013893__Ga0170528_1001 both contain integrases, phage repressor proteins, and the first has a tRNA-*Ala* adjacent the integrase, a potential host integration site. **b** Synteny between 4 putative phage / prophage sequences. Note the tRNA, putative integration site adjacent to one of two Terminal inverted repeat sequences, designated by the blue vertical bars. The "ANCHOR" gene was the gene used to center both plots.

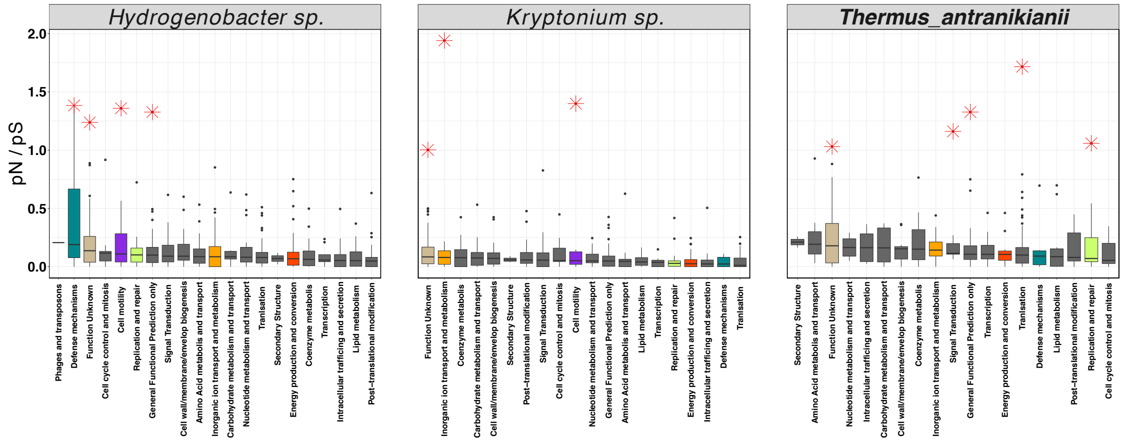

Supplementary Figure 6. pN/pS boxplots grouped by COG category. COG categories of interest are colored, and genes with pN/pS > 1 are noted by the red stars, as these are genes that may be under selection.

Population	Reference Completeness (%)	Genome size (Mb)	SNPs	% Polymorphic	NonSyn/kb	Syn/kb	Nuc div
Hydrogenobacter sp.	99	1.7	22810	1.36	4.4	42	2.0
Kryptonium sp.	96	2.6	23866	0.92	1.7	26	1.5
Thermus_antranikianii	71	1.4	8571	0.61	7.2	50	1.8

Supplementary Table 1. SNP statistics for each of the three analyzed populations.

References

1. Parks DH, Rinke C, Chuvochina M, Chaumeil P-A, Woodcroft BJ, Evans PN, et al. Recovery of nearly 8,000 metagenome-assembled genomes substantially expands the tree of life. *Nat Microbiol* 2017; 2: 1533–1542.
2. Nawrocki EP, Eddy SR. Infernal 1.1: 100-fold faster RNA homology searches. *Bioinformatics* 2013; 29: 2933.
3. Nawrocki EP, Burge SW, Bateman A, Daub J, Eberhardt RY, Eddy SR, et al. Rfam 12.0: updates to the RNA families database. *Nucleic Acids Res* 2015; 43: D130.
4. Walters WA, Caporaso JG, Lauber CL, Berg-Lyons D, Fierer N, Knight R. PrimerProspector: de novo design and taxonomic analysis of barcoded polymerase chain reaction primers. *Bioinformatics* 2011; 27: 1159.
5. Eloe-Fadrosh EA, Ivanova NN, Woyke T, Kyrpides NC. Metagenomics uncovers gaps in amplicon-based detection of microbial diversity. *Nat Microbiol* 2016; 1: 15032.
6. Katoh K, Standley DM. MAFFT Multiple Sequence Alignment Software Version 7: Improvements in Performance and Usability. *Mol Biol Evol* 2013; 30: 772.
7. Capella-Gutiérrez S, Silla-Martínez JM, Gabaldón T. trimAl: a tool for automated alignment trimming in large-scale phylogenetic analyses. *Bioinformatics* 2009; 25: 1972.
8. Nguyen L-T, Schmidt HA, von Haeseler A, Minh BQ. IQ-TREE: a fast and effective stochastic algorithm for estimating maximum-likelihood phylogenies. *Mol Biol Evol* 2015; 32: 268–74.
9. Markowitz VM, Chen I-MA, Chu K, Szeto E, Palaniappan K, Pillay M, et al. IMG/M 4 version of the integrated metagenome comparative analysis system. *Nucleic Acids Res* 2014; 42: D568-73.
10. Li W, Godzik A. Cd-hit: a fast program for clustering and comparing large sets of protein or nucleotide sequences. *Bioinformatics* 2006; 22: 1658–1659.
11. Chen IMA, Chu K, Palaniappan K, Ratner A, Huang J, Huntemann M, et al. The IMG/M data management and analysis system v.6.0: New tools and advanced capabilities. *Nucleic Acids Res* 2021; 49: D751–D763.
12. Yu G, Smith DK, Zhu H, Guan Y, Lam TT-Y. ggtree : an r package for visualization and annotation of phylogenetic trees with their covariates and other associated data. *Methods Ecol Evol* 2017; 8: 28–36.
13. Bushnell B, Rood J, Singer E. BBMerge – Accurate paired shotgun read merging via overlap. *PLoS One* 2017; 12: e0185056.
14. Chaumeil PA, Mussig AJ, Hugenholtz P, Parks DH. GTDB-Tk: A toolkit to classify genomes with the genome taxonomy database. *Bioinformatics* 2020; 36: 1925–1927.
15. Brown CT, Hug LA, Thomas BC, Sharon I, Castelle CJ, Singh A, et al. Unusual biology across a group comprising more than 15% of domain Bacteria. *Nature* 2015; advance on.
16. Yarza P, Yilmaz P, Pruesse E, Oliver Glöckner F, Ludwig W, Schleifer K-H, et al. Uniting the classification of cultured and uncultured bacteria and archaea using 16S rRNA gene sequences. *Nat Publ Gr* 2014; 12.
17. Jain C, Rodriguez-R LM, Phillippy AM, Konstantinidis KT, Aluru S. High-throughput ANI Analysis of 90K Prokaryotic Genomes Reveals Clear Species Boundaries. *bioRxiv* 2017; 225342.
18. Enright AJ, Dongen S Van, Ouzounis CA. An efficient algorithm for large-scale detection of

protein families. *Nucleic Acids Res* 2002; 30: 1575.
19. Klappenbach JA, Goris J, Vandamme P, Coenye T, Konstantinidis KT, Tiedje JM. DNA–DNA hybridization values and their relationship to whole-genome sequence similarities. *Int J Syst Evol Microbiol* 2007; 57: 81–91.
20. Emms DM, Kelly S. OrthoFinder: solving fundamental biases in whole genome comparisons dramatically improves orthogroup inference accuracy. *Genome Biol* 2015; 16: 157.
21. Nayfach S, Rodriguez-Mueller B, Garud N, Pollard KS. An integrated metagenomics pipeline for strain profiling reveals novel patterns of bacterial transmission and biogeography. *Genome Res* 2016; 26: 1612–1625.
22. Kurtz S, Phillippy A, Delcher AL, Smoot M, Shumway M, Antonescu C, et al. Versatile and open software for comparing large genomes. *Genome Biol* 2004; 5.
23. Tonkin-Hill G, Lees JA, Bentley SD, Frost SDW, Corander J. RhierBAPs: An R implementation of the population clustering algorithm hierbaps [version 1; referees: 2 approved]. *Wellcome Open Res* 2018; 3.
24. Wickham H. ggplot2: Elegant Graphics for Data Analysis. 2016. Springer-Verlag New York.
25. Bowers RM, Kyrpides NC, Stepanauskas R, Harmon-Smith M, Doud D, Reddy TBK, et al. Minimum information about a single amplified genome (MISAG) and a metagenome-assembled genome (MIMAG) of bacteria and archaea. *Nat Biotechnol* 2017; 35: 725–731.
